# Treating Presymptomatic Spinal Muscular Atrophy Patients with Onasemnogene Abeparvovec in Italy: The Role of the National Health System and Drug Supply. Comment on Zaidman et al. Newborn Screening for Spinal Muscular Atrophy: Variations in Practice and Early Management of Infants with Spinal Muscular Atrophy in the United States. *Int. J. Neonatal Screen.* 2024, *10*, 58

**DOI:** 10.3390/ijns11040102

**Published:** 2025-10-31

**Authors:** Riccardo Masson, Serena Gaballo, Raffaella Caravita, Stefano Parravicini

**Affiliations:** 1 Developmental Neurology Unit, Fondazione IRCCS Istituto Neurologico Carlo Besta, 20133 Milan, Italy; stefano.parravicini@istituto-besta.it; 2 Novartis Farma, 20154 Milan, Italy; serena.gaballo@novartis.com (S.G.); raffaella.caravita@gmail.com (R.C.)

We read with interest the recent study by Zaidman et al., which explores the early clinical management of infants diagnosed with spinal muscular atrophy (SMA) through newborn screening (NBS) across the United States. This survey-based observational study highlights critical challenges in the timely initiation of SMA therapy. Notably, 39% of providers administered treatment only after three weeks of age in NBS-positive infants, and in no cases was the treatment initiated within the first week of life. The primary barrier to early intervention was the prolonged insurance authorization process. Most providers (81%) considered gene therapy with onasemnogene abeparvovec (OA), the preferred first-line treatment [1].

NBS programs enable the early diagnosis of SMA at the presymptomatic or pauci-symptomatic stage, offering the most favorable window for early intervention to achieve motor development and minimize cumulative disability associated with motor impairment, respiratory and feeding complications [2,3,4,5,6,7]. In countries where NBS is implemented, access to therapy may vary depending on indication, treatment availability, and reimbursement eligibility criteria [8]. Prompt administration of pharmacological therapies is crucial, as their efficacy is greatest when patients receive treatment at the earliest possible stage [2,3,9]. Since OA cannot be stored by the provider, the role of the supply chain and its interaction with the healthcare system organization are critical to ensuring timely administration to patients [10].

Following the recent publication by Zaidman et al. [1], we present the Italian experience with the treatment pathway and timing of OA administration in infants diagnosed with SMA (data on file, Novartis). Unlike in the United States, private insurances are not involved in determining access to SMA therapy in Italy. After the genetic confirmation of positive SMA screening results through NBS, which also assesses *SMN2* copy number, eligibility to OA is evaluated based on both clinical and reimbursement criteria. Specifically, patients must carry up to 3 *SMN2* copies (Table 1), have a body weight of at least 2.6 kg and a negative anti-adeno-associated virus (AAV) 9 titer at the time of OA administration. To confirm eligibility, pre-dosing laboratory assessments should include liver function parameters (i.e., aspartate aminotransferase, alanine aminotransferase, total bilirubin, albumin), coagulation markers (prothrombin time, partial thromboplastin time), troponin-I and creatinine levels, and a complete blood count (including platelet count) [10]. Eligibility for treatment is confirmed through a digital prescription, ensuring that Italian Health Authority approval is granted at the time of the clinical assessment.

In 2024, 16 NBS-positive infants diagnosed with SMA in Italy received OA. Of these, 11 were treated immediately after the screening evaluation, while the remaining 5 patients received OA at a later stage (range: 39–162 days). Reasons for bridge therapies included being underweight or preterm, the need for further medical assessments, and a high level of anti-AAV9 antibodies.

In the immediately-treated group, therapy was administered at a mean age of 18.3 days (standard deviation [SD] 5.3) and a median age of 17 days (range: 10–27). Notably, more than a one-quarter of them (3 out of 11, 27.3%) received the infusion within the first two weeks of life, with the earliest treatment occurring at just 10 days of life.

These data underscore that, despite the extensive clinical and laboratory assessments required to confirm infant eligibility to OA, early initiation of therapy can be successfully achieved in patients identified as eligible immediately after NBS. Standardized laboratory procedures and accurate interpretation of genetic test results are essential prerequisites to prevent treatment deferral [12]. Furthermore, the integration of a digital prescription system and seamless coordination between treatment centers and the Novartis supply chain are crucial for ensuring timely access to gene therapy during the presymptomatic phase.

Conversely, when patients present concomitant conditions, such as preterm birth, low birth weight, infections or other pediatric diseases, OA treatment must be avoided or delayed. In these cases, children should be treated with an alternative drug and OA administration may be considered later. Infants with SMA, particularly those with only 2 *SMN2* copies, may develop symptoms rapidly, sometimes within the first days or weeks of life [12]. Therefore, delays in treatment initiation could significantly impact the therapeutic outcome, and the initiation of another disease-modifying therapy or a bridging strategy is mandatory when gene therapy eligibility cannot be confirmed at diagnosis [7,13,14,15].

In addition to medical reasons, logistical factors may adversely affect timelines in the Italian context, and they must be carefully managed to ensure timely OA administration. Given that gene therapy cannot be stored by the provider, once OA is selected as the treatment of choice, two crucial steps are essential to secure expedited delivery: (1) timely completion of the digital prescription by the physician, and (2) authorization of the purchase and prompt placement of the drug order by the pharmacist. Finally, poor caregiver compliance, family-related logistical issues, initial treatment refusal, and linguistic or cultural barriers, though uncommon, may also contribute to treatment delay.

Nevertheless, our experience demonstrates that the administration of OA within the first two weeks of life is feasible in most cases in Italy. The continuous and well-coordinated collaboration between treatment centers and the Novartis supply chain is essential for achieving this goal. This efficient process enables the administration of OA—when selected as the first choice—before symptoms onset.

In conclusion, variations in NBS pathways and access regulations for SMA therapies are key factors influencing the time from birth to treatment initiation worldwide [8]. The age at which OA is administered as the first-line treatment in Italy following NBS is currently satisfactory. Nevertheless, the process warrants continuous monitoring and systematic efforts for improvement across all sites nationwide. Achieving true equity in SMA care requires the identification and elimination of bottlenecks that delay early intervention, ensuring that every newborn, regardless of geographic location, has comparable access to timely, life-changing therapies.

## Figures and Tables

**Table 1:** Available SMA therapies in Italy according to *SMN2* copy number [11].

*SMN2* Copy Number	Therapy
<4	onasemnogene abeparvovec, risdiplam, nusinersen
≥4	risdiplam, nusinersen

## Data Availability

The original contributions presented in this study are included in the article. Further inquiries can be directed to the corresponding author.

## Abbreviations

The following abbreviations are used in this manuscript:

AAV	Adeno-Associated Virus
NBS	Newborn Screening
OA	Onasemnogene Abeparvovec
SD	Standard Deviation
SMA	Spinal Muscular Atrophy
SMN2	Survival Motor Neuron 2

## References

[1] Zaidman C.M., Crockett C.D., Wedge E., Tabatabai G., Goedeker N. (2024). Newborn Screening for Spinal Muscular Atrophy: Variations in Practice and Early Management of Infants with Spinal Muscular Atrophy in the United States. Int. J. Neonatal Screen..

[2] De Vivo D.C., Bertini E., Swoboda K.J., Hwu W.L., Crawford T.O., Finkel R.S., Kirschner J., Kuntz N.L., Parsons J.A., Ryan M.M. (2019). Nusinersen initiated in infants during the presymptomatic stage of spinal muscular atrophy: Interim efficacy and safety results from the Phase 2 NURTURE study. Neuromuscul. Disord..

[3] Strauss K.A., Farrar M.A., Muntoni F., Saito K., Mendell J.R., Servais L., McMillan H.J., Finkel R.S., Swoboda K.J., Kwon J.M. (2022). Onasemnogene abeparvovec for presymptomatic infants with three copies of SMN2 at risk for spinal muscular atrophy: The Phase III SPR1NT trial. Nat. Med..

[4] Weiss C., Becker L.L., Friese J., Blaschek A., Hahn A., Illsinger S., Schwartz O., Bernert G., Hagen M.V., Husain R.A. (2024). Efficacy and safety of gene therapy with onasemnogene abeparvovec in children with spinal muscular atrophy in the D-A-CH-region: A population-based observational study. Lancet Reg. Health Eur..

[5] Moultrie F., Chiverton L., Hatami I., Lilien C., Servais L. (2025). Pushing the boundaries: Future directions in the management of spinal muscular atrophy. Trends Mol. Med..

[6] Servais L., Day J.W., De Vivo D.C., Kirschner J., Mercuri E., Muntoni F., Proud C.M., Shieh P.B., Tizzano E.F., Quijano-Roy S. (2024). Real-World Outcomes in Patients with Spinal Muscular Atrophy Treated with Onasemnogene Abeparvovec Monotherapy: Findings from the RESTORE Registry. J. Neuromuscul. Dis..

[7] Schwartz O., Vill K., Pfaffenlehner M., Behrens M., Weiss C., Johannsen J., Friese J., Hahn A., Ziegler A., Illsinger S. (2024). Clinical Effectiveness of Newborn Screening for Spinal Muscular Atrophy: A Nonrandomized Controlled Trial. JAMA Pediatr..

[8] Armengol V.D., Darras B.T., Abulaban A.A., Alshehri A., Barisic N., Ben-Omran T., Bernert G., Castiglioni C., Chien Y.H., Farrar M.A. (2024). Life-Saving Treatments for Spinal Muscular Atrophy: Global Access and Availability. Neurol. Clin. Pract..

[9] Servais L., Farrar M., Finkel R., Vlodavets D., Zanoteli E., Al-Muhaizea M., de Queiroz Campos Araújo A.P., Nelson L., Jaber B., Gorni K. (2024). RAINBOWFISH: Primary Efficacy and Safety Data in Risdiplam-treated Infants with Presymptomatic Spinal Muscular Atrophy (SMA) (S37.006). Neurology.

[10] EMA Zolgensma Summary of Product Characteristics. https://www.ema.europa.eu/en/documents/product-information/zolgensma-epar-product-information_en.pdf.

[11] Ambrosio A., Fioretti T., D’Andrea B., Pezone L., Bitetti I., Di Domenico C., Vallone S., Maiolo V., Cioce A., Giustino M. (2025). Newborn Screening Program for Spinal Muscular Atrophy in the Campania Region (Italy): Current Limitations and Potential Perspectives. Int. J. Neonatal Screen..

[12] Aragon-Gawinska K., Mouraux C., Dangouloff T., Servais L. (2023). Spinal Muscular Atrophy Treatment in Patients Identified by Newborn Screening-A Systematic Review. Genes.

[13] Proud C.M., Mercuri E., Finkel R.S., Kirschner J., De Vivo D.C., Muntoni F., Saito K., Tizzano E.F., Desguerre I., Quijano-Roy S. (2023). Combination disease-modifying treatment in spinal muscular atrophy: A proposed classification. Ann. Clin. Transl. Neurol..

[14] Finkel R.S., Benatar M. (2022). Pre-symptomatic spinal muscular atrophy: A proposed nosology. Brain.

[15] Cooper K., Nalbant G., Sutton A., Harnan S., Thokala P., Chilcott J., McNeill A., Bessey A. (2024). Systematic Review of Presymptomatic Treatment for Spinal Muscular Atrophy. Int. J. Neonatal Screen..

